# Second Generation mTOR Inhibitors as a Double-Edged Sword in Malignant Glioma Treatment

**DOI:** 10.3390/ijms20184474

**Published:** 2019-09-10

**Authors:** Dennis Heinzen, Iris Divé, Nadja I. Lorenz, Anna-Luisa Luger, Joachim P. Steinbach, Michael W. Ronellenfitsch

**Affiliations:** 1 Dr. Senckenberg Institute of Neurooncology, University Hospital Frankfurt, Schleusenweg 2-16, 60528 Frankfurt am Main, Germany; 2 University Cancer Center Frankfurt (UCT), University Hospital Frankfurt, Theodor-Stern-Kai 7, 60590 Frankfurt am Main, Germany; 3 German Cancer Consortium (DKTK), Partner Site Frankfurt/Mainz, Theodor-Stern-Kai 7, 60590 Frankfurt am Main, Germany; 4 Frankfurt Cancer Institute (FCI), University Hospital Frankfurt, Paul-Ehrlich-Straße 42-44, 60596 Frankfurt am Main, Germany

**Keywords:** glioblastoma, mTOR, mTOR inhibition, hypoxia, starvation, tumor microenvironment

## Abstract

Glioblastomas (GBs) frequently display activation of the epidermal growth factor receptor (EGFR) and mammalian target of rapamycin (mTOR). mTOR exists as part of two multiprotein complexes, mTOR complex 1 (mTORC1) and 2 (mTORC2). In GBs, mTORC1 inhibitors such as rapamycin have performed poorly in clinical trials, and in vitro protect GB cells from nutrient and oxygen deprivation. Next generation ATP-competitive mTOR inhibitors with affinity for both mTOR complexes have been developed, but data exploring their effects on GB metabolism are scarce. In this study, we compared the ATP-competitive mTORC1/2 inhibitors torin2, INK-128 and NVP-Bez235 to the allosteric mTORC1 inhibitor rapamycin under conditions that mimic the glioma microenvironment. In addition to inhibiting mTORC2 signaling, INK-128 and NVP-Bez235 more effectively blocked mTORC1 signaling and prompted a stronger cell growth inhibition, partly by inducing cell cycle arrest. However, under hypoxic and nutrient-poor conditions mTORC1/2 inhibitors displayed even stronger cytoprotective effects than rapamycin by reducing oxygen and glucose consumption. Thus, therapies that arrest proliferation and inhibit anabolic metabolism must be expected to improve energy homeostasis of tumor cells. These results mandate caution when treating physiologically or therapeutically induced hypoxic GBs with mTOR inhibitors.

## 1. Introduction

Despite significant advances in molecular tumor characterization, and increasing knowledge on tumor biology, the prognosis of patients diagnosed with glioblastoma (GB) remains poor [1]. The epidermal growth factor receptor (EGFR) is part of the most frequently altered signal transduction cascade in GB [2]. EGFR signaling leads to the activation of downstream targets including phosphoinositide 3-kinase (PI3K) and mammalian target of rapamycin (mTOR), which exists as part of two major signaling complexes termed mTORC1 and mTORC2 [3]. A key regulator of cellular metabolism, mTORC1 induces protein and lipid synthesis as well as cell growth. By phosphorylating the eukaryotic translation initiation factor 4E binding protein 1 (4EBP1), and the ribosomal S6 kinase 1 (S6K1), which in turn phosphorylates the S6 ribosomal protein (S6RP), mTORC1 activates a downstream cascade that leads to increased protein translation via factors such as the eukaryotic translation initiation factor 4E (eIF4E) (Figure S1) [4,5]. Another downstream target of mTORC1 is the UNC-51-like kinase 1 (ULK1). By phosphorylating ULK1, mTORC1 inhibits one of the key components of the autophagy initiating complex [6]. The substrates of mTORC2 include Akt and members of the serine/threonine-protein kinases (SGK) family. An important downstream target of mTORC2 is the n-myc downstream regulated gene 1 (NDRG1) which has been implicated to play a role in cancer metastasis [7] and confer resistance to chemotherapy in malignant glioma [8].

Genomic analyses of human GBs have revealed that genetic alterations of the EGFR exist in up to 57% of patient samples [9], including deletion or missense mutation in the extracellular receptor domains that cause ligand-independent signaling [10,11]. Found in approximately 36% of tumors, mutations in the tumor suppressor phosphatase and tensin homolog (PTEN) further increase downstream signaling activity [2]. Given its central function and position downstream of EGFR and PTEN, mTOR is a plausible target for therapeutic inhibition. Indeed, pharmacological inhibition of EGFR and mTOR had antiproliferative effects in glioma cells in vitro, and in some studies synergized with chemotherapy or radiation [12,13,14]. Yet, clinical trials with mTORC1 inhibitors produced largely disappointing results [3]. In the recent RTOG-0913 phase II trial, addition of the rapamycin-derivative everolimus to standard radio- and chemotherapy in patients with newly diagnosed GBs resulted in a significantly reduced overall survival and was associated with severe toxicity and mortality [15]. While no convincing explanation for this effect was offered [16], we have previously reported that survival in cells exposed to hypoxia is enhanced by EGFR and mTOR inhibition [17,18,19]. In line with this, mTORC1 activation sensitized human glioma cells to hypoxia-induced cell death by increasing oxygen consumption and ATP depletion [20]. Since hypoxic and nutrient-deprived areas dominate the glioma microenvironment, pharmacological mTOR inhibition could ultimately generate pro-survival effects in tumor cells.

Importantly, most of the above-mentioned in vivo and in vitro studies used mTOR inhibitors that are derivatives of the original rapamycin compound (so-called rapalogs) acting via allosteric mTORC1 inhibition [21]. Since incomplete suppression of the mTOR signaling pathway could be a reason for drug failure, ATP-competitive mTOR inhibitors have been developed with the ability to more effectively inhibit mTORC1, and to additionally inhibit mTORC2. To date, the question whether these novel mTORC1/2 inhibitors could generate antitumor effects superior to those of rapalogs has not been addressed for GB cells under conditions of the tumor microenvironment. Hence, in this project we compared novel ATP-competitive mTORC1/2 inhibitors to rapamycin with regard to their capacity to affect glioma cell metabolism and growth. Specifically, we investigated the effects of torin2 and INK-128, which both act as dual mTORC1/2 inhibitors [22,23]. Of note, torin2 also shows activity against the ataxia telangiectasia mutated kinase (ATM) and the ataxia telangiectasia and Rad-3-related kinase (ATR) [24]. Additionally, we tested NVP-Bez235, which inhibits mTORC1/2 as well as PI3K [25], and has been shown to sensitize glioma cells to chemotherapy and radiation [26]. We hypothesized that the dual complex specificity as well as the more complete target inhibition of mTORC1 could help overcome the limitations of mTORC1 inhibitors. We here report that, indeed, mTORC1/2 inhibitors are more potent in inhibiting GB cell growth than mTORC1-specific rapalogs. However, in line with our previous results, ATP-competitive mTOR inhibitors profoundly affected cellular metabolism leading to increased tolerance to nutrient deprivation and hypoxia. Our results highlight the central role of mTOR as a metabolic regulator of cell proliferation and energy homeostasis.

## 2. Results

### 2.1. INK-128, Torin2 and NVP-Bez235 Effectively Inhibit Phosphorylation of mTOR Downstream Targets S6RP and NDRG1

**Figure 1:**
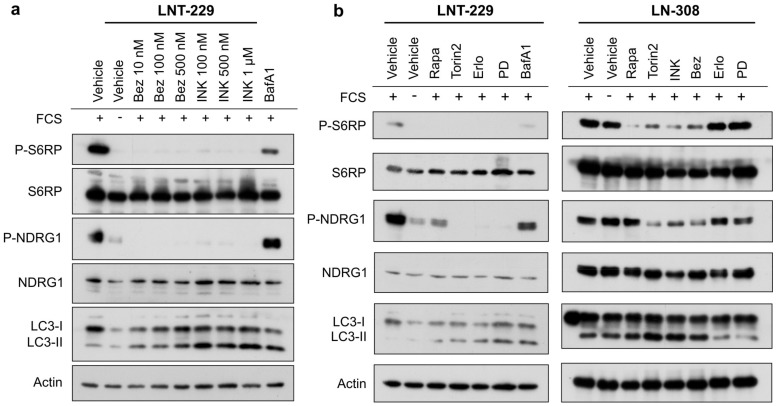
ATP-competitive mammalian target of rapamycin (mTOR) inhibitors effectively inhibit mTOR complex 1 (mTORC1) and 2 (mTORC2) signal transduction. (**a**) LNT-229 cells were treated with INK-128, NVP-Bez235 or bafilomycin A1 (BafA1) (10 nM) as depicted in medium containing 10% fetal calf serum (FCS) for 2 h. Cell lysates were analyzed by immunoblot for phosphorylated (P) and total mTORC1 target protein S6RP and mTORC2 target protein NDRG1. (**b**) LNT-229 and LN-308 cells were treated for 2 h with rapamycin (100 nM), torin2 (100 nM), INK-128 (100 nM), NVP-Bez235 (10 nM), erlotinib (10 µM), PD153035 (10 µM) or bafilomycin A1 (BafA1) (10 nM) in FCS-free medium or medium containing 10% FCS as indicated.

Since the effects of INK-128 and NVP-Bez235 on human glioma cells have been characterized only marginally, we first assessed their capacity to effectively inhibit mTOR signaling in LNT-229 glioma cells at different concentrations. INK-128 and NVP-Bez235 sufficiently inhibited phosphorylation of mTOR target proteins NDRG1 and S6RP at concentrations of 100 and 10 nM, respectively (Figure 1a). This coincided with the conversion of microtubule-associated protein 1A/1B-light chain three-I (LC3-I) to LC3-II, an indicator for the initiation of autophagy by mTOR inhibition. Based on these findings, the concentrations of INK-128 100 nM and NVP-Bez235 10 nM were set for further analysis.

Regarding their ability to inhibit mTOR signaling, we next compared INK-128, torin2 and NVP-Bez235 to rapamycin, as well as to the EGFR inhibitors PD153035 and erlotinib. Concentrations of rapamycin, torin2, PD153035 and erlotinib were chosen based on previous studies [18]. INK-128, torin2 and NVP-Bez235 were similarly efficient to inhibit mTOR signaling from both complexes (Figure 1b). As expected, rapamycin reduced phosphorylation of NDRG1 to a much lesser extent than dual mTORC1/2 inhibitors. Similar results were obtained in G55 cells (Figure S2). Importantly, INK-128, torin2 and NVP-Bez235 displayed a higher efficacy in blocking phosphorylation of 4EBP1 (Figure S3). The limited efficacy of EGFR inhibitors in LN-308 was expected given the PTEN mutation in this cell line (Figure 1b, right panel) [27].

### 2.2. ATP-Competitive mTORC1/2 Inhibitors More Potently Inhibit Glioma Cell Growth by Inducing Cell Cycle Arrest

**Figure 2:**
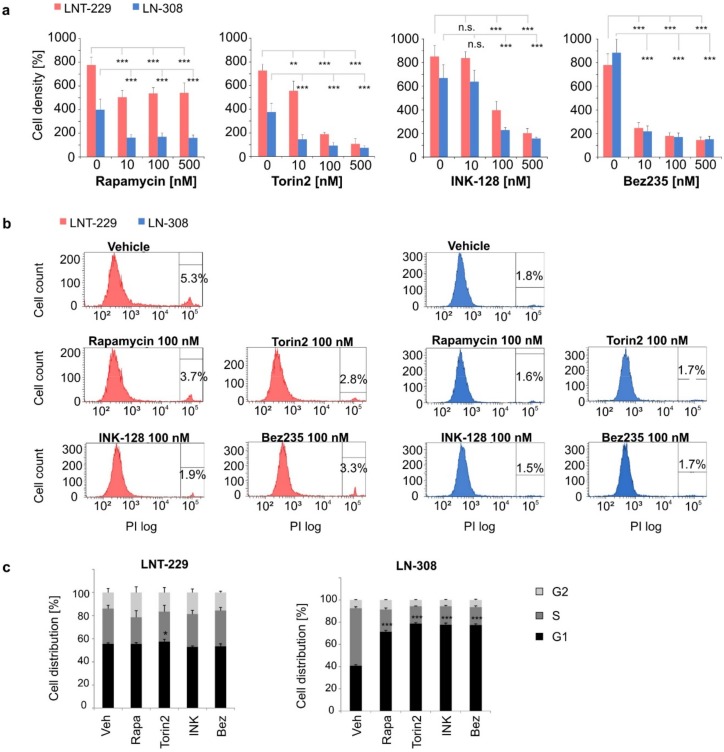
Inhibition of mTORC1 and 2 reduces glioma cell growth. (**a**) LNT-229 and LN-308 cells were incubated in medium containing 10% FCS and treated with rapamycin, torin2, INK-128 or NVP-Bez235 at indicated concentrations for 72 h. Cell density was analyzed by crystal violet staining. Depicted values are normalized to cell density at the beginning of treatment (*n* = 3, mean ± S.D.; **p < 0.01, ***p < 0.001, n.s. = not significant). (**b**) Cytotoxicity of the indicated inhibitor was assessed by propidium iodide (PI) staining and consecutive FACS analysis after incubation for 72 h as described in (**a**) (one representative analysis of *n* = 3). (**c**) For cell cycle analysis, LN308 or LNT-229 cells were treated with the indicated mTOR inhibitor for 24 h (100 nM rapamycin, 100 nM torin2, 100 nM INK-128 or 10 nM NVP-Bez235). DNA content as a marker of cell cycle phase was measured by PI staining after permeabilization using flow cytometry. The depicted values correspond to the quantification of relative cell cycle phase distribution (*n* = 3, mean ± S.D., *p < 0.05, ***p < 0.001).

To assess the impact of mTOR inhibition on cell growth we treated glioma cell lines with NVP-Bez235, INK-128, torin2, rapamycin (Figure 2a), erlotinib or PD153035 (Figure S4a). The panel of LNT-229, LN-308 and G55 cells was chosen because it covers the molecular spectrum of typical GBs with differing p53, PTEN and MGMT promoter methylation status (Table 1). INK-128, NVP-Bez235 and torin2 caused a dose-dependent growth inhibition in serum-containing conditions that was superior to the effect of rapamycin. Treatment with mTOR inhibitors did not coincide with cytotoxicity (Figure 2b). EGFR inhibitors, however, showed different degrees of dose-dependent cytotoxicity (Figure S4b). Since induction of G1 arrest following rapamycin treatment has been reported [28], we performed cell cycle analysis. Cell cycle arrest was detectable in LN-308 cells after treatment with mTOR inhibitors (Figure 2c). Notably, the more potent growth inhibition by INK-128 and NVP-Bez235 correlated with a larger G1 subgroup. This effect was less pronounced in LNT-229 (Figure 2c) and G55 cells (Figure S5).

### 2.3. mTOR Inhibition Reduces Oxygen and Glucose Consumption in Glioma Cells

**Figure 3:**
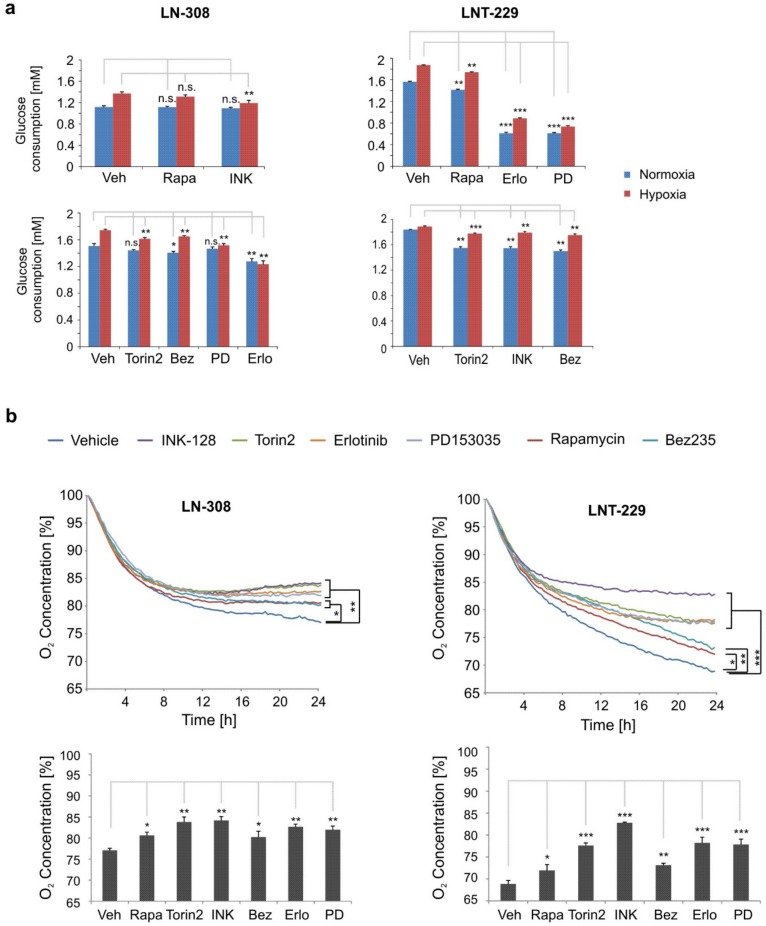
mTOR inhibition attenuates glucose and oxygen consumption. (**a**) LNT-229 and LN-308 cells were cultured in medium containing 2 mM glucose and treated with rapamycin (100 nM), torin2 (100 nM), INK-128 (100 nM), NVP-Bez235 (10 nM), erlotinib (10 µM) or PD153035 (10 µM) in hypoxia (0.1% oxygen) for 8 h. Glucose consumption was measured via quantification of remaining glucose in the supernatant (*n* = 3, mean ± S.D., *p < 0.05, **p < 0.01, ***p < 0.001, n.s. = not significant). (**b**) LNT-229 and LN-308 cells were exposed to serum-free medium with 25 mM glucose and treated with rapamycin (100 nM), torin2 (100 nM), INK-128 (100 nM), NVP-Bez235 (10 nM), erlotinib (10 µM) or PD153035 (10 µM). Oxygen consumption was measured with a fluorescence-based assay (*n* = 3, mean; *p < 0.05, **p < 0.01, ***p < 0.001). The end point analysis is depicted in the column charts (*n* = 3, mean ± S.D., *p < 0.05, **p < 0.01, ***p < 0.001).

The potential to economize cellular nutrient resources and thus to maintain energy homeostasis is most likely a major influence for enhanced cell survival under the nutrient and oxygen-deprived conditions of the tumor microenvironment. Since glucose is of particular relevance to tumor cells as an energy source [29], we measured glucose concentration in the supernatant of LNT-229 and LN-308 cells exposed to NVP-Bez235, torin2 or INK-128. All three inhibitors reduced glucose consumption in normoxia and hypoxia. These findings were consistent in G55 cells (Figure S6a). In comparison, rapamycin had only minor effects (Figure 3a). An overall stronger reduction of glucose consumption was achieved by the EGFR inhibitors PD153035 and erlotinib, most likely due to the EGFR-mediated regulation of the AKT/GLUT1 pathway [30,31]. In LN-308 cells, the effects of mTOR and EGFR inhibitors were less prominent in normoxia, but more pronounced in hypoxia. We then asked whether corresponding changes in oxygen consumption could be detected. NVP-Bez235, torin2 and INK-128 significantly decreased oxygen consumption in LNT-229 and LN-308 as indicated by a higher remaining oxygen concentration in the respective culture medium (Figure 3b). INK-128 appeared most potent, and in LNT-229 cells caused the largest decrease in oxygen consumption of the inhibitors. In this regard, the results produced by NVP-Bez235 treatment were comparable to rapamycin, but inferior to torin2. PD153035 and erlotinib decreased oxygen consumption in glioma cells. However, the extent of this effect was cell line dependent. In G55 cells, treatment with PD153035 and erlotinib did not result in a significant reduction of oxygen consumption (Figure S6b).

### 2.4. INK-128, Torin2 and NVP-Bez235 Protect Human Glioma Cells from Hypoxia-Induced Cell Death

**Figure 4:**
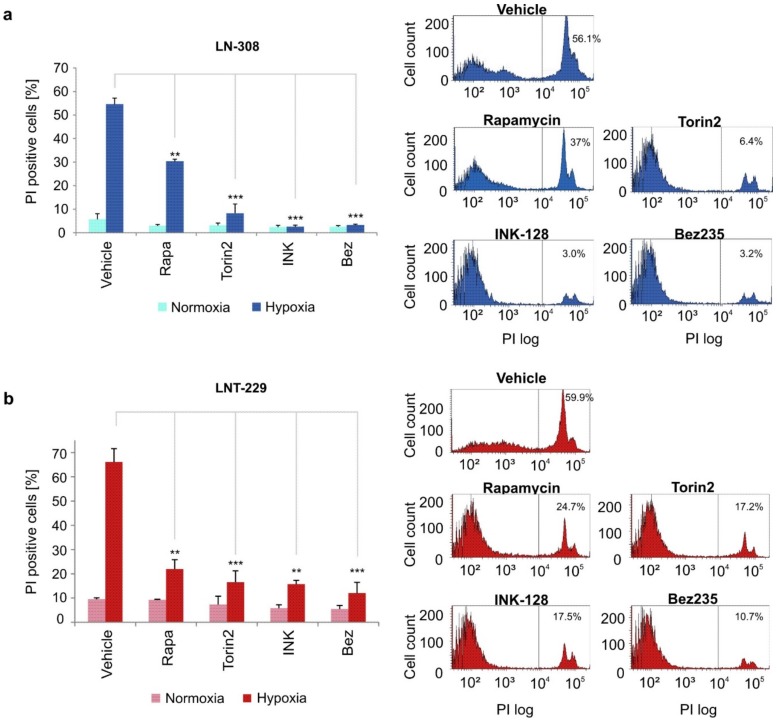
ATP-competitive mTOR inhibitors protect human glioma cells from hypoxia-induced cell death. LN-308 (**a**) and LNT-229 cells (**b**) were exposed to serum-free medium with 2 mM glucose and hypoxia (0.1% oxygen) and treated with rapamycin (100 nM), torin2 (100 nM), INK-128 (100 nM) or NVP-Bez235 (10 nM). Treatment duration was approximately 22 h for LN-308 and 26 h for LNT-229 cells. Cell death was then quantified by PI staining (*n* = 3, mean ± S.D., **p < 0.01, ***p < 0.001).

Based on our findings that torin2, NVP-Bez235 and INK-128 reduce glucose and oxygen consumption, we hypothesized that these metabolic changes could entail a survival advantage for glioma cells in starvation conditions. Indeed, when tested under glucose-restricted and hypoxic experimental conditions, all mTOR inhibitors protected LNT-229 and LN-308 from cell death (Figure 4a,b). Notably, mTORC1/2 inhibitors were much more potent in reducing the percentage of PI positive cells than rapamycin. In LN-308 cells, hypoxia-induced cell death was largely abolished. Dose-response curves were performed in LNT-229 cells and indicated that the concentrations chosen corresponded to the maximum effect range for each substance without any additional effect at higher concentrations (Figure S7).

To account for the mutational landscape of malignant gliomas, we sought to confirm these effects in glioma cell lines with differing mutational profiles and growth characteristics. We additionally investigated the effects of mTOR inhibition in glioma cell lines G55 and T98G. Similar results were obtained in G55 cells (Figure S8a). In the glioma cell line T98G, which showed overall high sensitivity towards mTOR inhibition, the effects of rapamycin and mTORC1/2 kinase inhibitors did not differ significantly (Figure S8b). We additionally tested two cell lines from other cancer entities, including HCT-116 colon carcinoma cells and MDA-MB-231 breast cancer cells (Figure S8c,d). While mTOR inhibition did enhance cell survival under hypoxia in these tumor cells, these effects were overall less pronounced. However, significant changes in cell survival were observed with ATP-competitive mTOR inhibitors only.

## 3. Discussion

Considering their central role in cell metabolism and their frequent activation due to genetic alterations, EGFR and mTOR are plausible targets for therapeutic inhibition in GB. Results from clinical trials, however, argue against the commonly employed approach of simply adding an inhibitor to standard treatment in unselected cohorts. Everolimus, an allosteric mTOR inhibitor, even produced detrimental effects leading to shorter overall survival in the RTOG 0913 trial [15]. In this context, our study highlights the profound metabolic effects of signal transduction inhibition that can promote tumor cell survival under realistic conditions of the glioma microenvironment [32] and provides a potential explanation for the poor performance of everolimus in the RTOG 0913 trial. However, it is noteworthy that everolimus has been employed successfully in the treatment of gliomas, in particular subependymal giant cell astrocytomas (SEGAs) [33]. SEGAs are tumors frequently arising in patients with tuberous sclerosis complex that have activated mTORC1 signaling due to mutations frequently affecting the TSC1 or TSC2 genes (Figure S1) [34]. In contrast to GBs, SEGAs are WHO grade I benign tumors that do not show areas of necroses or hypoxia [20]. Therefore, the potentially protective metabolic effects of mTOR inhibition appear less relevant in this entity.

The development of small-molecule substances that inhibit both mTORC1 and mTORC2 has sparked new hope to successfully integrate mTOR inhibition into GB treatment. In this study, we hypothesized that a more complete target inhibition could help overcome the protective metabolic effects of mTOR inhibition observed in previous studies [18,20]. Indeed, mTORC1/2 inhibitors torin2, NVP-Bez235 and INK-128 showed a more complete mTORC1 pathway suppression evidenced by reduced phosphorylation of 4EBP1 (Figure S2A). As expected, they also blocked mTORC2 signaling. In comparison with rapamycin, dual mTORC1/2 inhibition led to stronger growth inhibition, which partly coincided with cell cycle arrest. Similar findings have been reported previously in LNT-229 and U87MG glioblastoma cell lines [35]. However, not only did mTORC1/2 inhibitors fail to cause cytotoxicity, they also showed a far greater ability to enhance glioma cell survival under hypoxic conditions. The fact that this coincided with reduced glucose and oxygen consumption indicates that the protection from cell death is mediated at least in part by global inhibition of metabolic activity. In this regard, enhanced cell survival by treatment with rapamycin or NVP-Bez235 was also observed under glucose restriction without additional hypoxia (Figure S9). Interestingly, mTORC1-mediated inhibitory phosphorylation of superoxide dismutase 1 (SOD1) have recently been reported to regulate reactive oxygen species (ROS) [36] which might offer additional protection under stress conditions. Accordingly, mTOR inhibitors reduced ROS levels in glioma cell lines as observed in an exploratory approach (Figure S10). From a translational perspective, mTORC1/2 inhibitors could efficiently target rapidly dividing glioma cells. When exposed to a nutrient-poor and hypoxic environment, however, tumor cells could benefit from metabolic changes induced by mTOR inhibitors that would ultimately enable them to better economize resources. The clinical implementation of mTORC1/2 inhibitors could be further limited by recently identified resistance mechanisms. The cultivation of breast cancer cells with AZD8055 caused the emergence of AZD8055-resistant clones that harbored increased mTOR kinase activity due to single amino acid mutations in the mTOR kinase domain that led to increased kinase activity [35]. Recently, attention was drawn to the role of mitochondria in conveying resistance to mTOR inhibition [37]. MTOR inhibition by the ATP-competitive kinase inhibitors INK-1341 and INK-128 induced profound changes in mitochondrial morphology and activated the mitochondrial hyperfusion reaction. Notably, these effects were less pronounced with rapamycin, similar to our results. Mechanistically, it was shown that mTORC1 inhibition led to translation of the mitochondrial fission process 1 (MTFP1). This was mediated by eIF4E-binding proteins, the phosphorylation of which is more efficiently blocked by mTOR kinase inhibitors than by rapamycin and its derivatives (Figure S3). The disruption of this mechanism turned the cytostatic effect of mTOR inhibition into cytotoxic.

In this light, it seems discussible to discard mTOR inhibition as a treatment option for malignant glioma. However, successful employment of targeted therapy approaches in other cancer entities required the identification of a true oncogenic driver. Two prominent examples thereof are the bcr–abl fusion gene which takes a central role in the pathogenesis of chronic myelogenous leukemia [38], as well as NTRK gene fusions occurring in a wide range of tumor types [39]. In this regard, results from the EORTC 26082 trial suggest that a subgroup of glioblastoma patients might be eligible for treatment with mTOR inhibitors. A phase II trial, it compared radiochemotherapy with temozolomide to radiotherapy and temsirolimus in patients with newly diagnosed glioblastoma without MGMT promoter methylation. While overall survival did not differ between the two groups, the presence of Ser2448 phosphorylated mTOR (an indicator of mTORC1 signaling activity [40]) in tissue samples as evaluated by immunohistochemistry correlated with better overall survival in the temsirolimus subgroup [41]. Recently, the German N^2^M^2^ trial of molecularly matched targeted therapies for patients with newly diagnosed GB without MGMT promoter methylation was initiated. Following molecular analysis of tumor tissue samples, patients with phosphorylated mTOR-Ser2448 will receive temsirolimus in addition to standard radiotherapy [42,43]. Similarly, an analysis of mTOR activation markers allowed the identification of a GB patient subgroup that might benefit from an EGFR-targeting therapy [44].

In sum, despite their superiority regarding the inhibition of 4EBP1 phosphorylation and dual affinity for both mTOR complexes, our results characterize mTORC1/2 inhibitors in vitro as a double-edged sword leading to a favorable growth inhibition on the one hand and an unfavorable protection from hypoxia-related cell death on the other. Taken together, our results highlight the challenges to successfully incorporate mTOR inhibitors into the treatment regimen for GB and underline the importance of pre-clinical experimental settings that adequately mirror the conditions of the tumor microenvironment in order to assess the potential of pharmacological inhibitors as a therapeutic option. As in other tumor entities, a rational subgroup selection and combination or a sequence treatment algorithm might be the key for the successful employment of mTOR inhibitors in GB treatment.

## 4. Materials and Methods

### 4.1. Reagents and Cell Lines

Reagents included rapamycin and torin2 (both Sigma, St. Louis, MO, USA), INK-128 and NVP-Bez235 (both Cayman Chemical Company, Ann Arbor, MI, USA), erlotinib (Sequoia Research Products, Pangbourne, UK), PD153035 and bafilomycin A1 (both Tocris, Minneapolis, MN, USA). LN-308 and LNT-229 were a kind gift from Dr. N. de Tribolet (Lausanne, Switzerland). G55 cells were a kind gift from Manfred Westphal and Kathrin Lamszus (Hamburg, Germany). The genetic background of the cell lines regarding *O*-6-methylguanine-DNA methyltransferase (MGMT), isocitrate dehydrogenase 1 (IDH1), p53 (TP53) and PTEN are shown below (Table 1) [20,27,45]. T98G cells were obtained from ATCC (Rockville, MD, USA). Human HCT116 colon carcinoma cells were kindly provided by B. Vogelstein. MDA-MB-231 cells were a kind gift from Winfried Wels (Frankfurt, Germany). Cells were maintained at 37 °C and 5% CO_2_ using Dulbecco’s modified eagle medium (DMEM) supplemented with 100 IU/mL penicillin, 100 µg/mL streptomycin (Life Technologies, Karlsruhe, Germany) and 10% fetal calf serum (Biochrom KG, Berlin, Germany).

### 4.2. Immunoblot

Cells were harvested and protein concentration was determined using the Bio-Rad Protein Assay (Bio-Rad, Munich, Germany). We subjected 20 µg acquired protein to SDS page analysis. After washing with tris-buffered saline with Tween20 and blocking in 5% skim milk, the resulting membranes were incubated overnight with antibodies to NDRG1, P-NDRG1 (Thr346), S6RP, P-S6RP (Ser240/44), 4EBP1, P-4EBP1, p62, p-ULK (all from Cell Signaling, Cambridge, UK) and LC-3 (Sigma). Following one hour of incubation with secondary rabbit or goat antibody (Santa Cruz Biotechnology, Dallas, TX, USA), chemiluminescence was used for detection.

### 4.3. Cell Density and Viability Assay

For cell growth assays, 20,000 cells were seeded in 96-well plates 24 h prior to treatment. Cell density was quantified by crystal violet (CV) staining as described before [46]. For glucose and oxygen consumption as well as hypoxia-induced cell death, cells were preincubated with the indicated inhibitors in serum-free medium. The effects of brief preincubation on cell density were ruled out by CV staining (Figure S11). Cell viability was assessed by propidium iodide (PI) uptake via flow cytometry as previously described [18]. For the assessment of hypoxia-induced cell death, cells were seeded in 24-well plates 24 h prior to treatment and were preincubated with the indicated inhibitors diluted in serum-free medium for 4 h. Treatment conditions were then changed to serum-free medium with 2 mM glucose containing the indicated inhibitors. Hypoxic conditions with 0.1% oxygen were achieved by use of Gas Pak pouches (Becton-Dickinson, Heidelberg, Germany). Cell viability was quantified by PI uptake after 22–40 h, depending on cell line.

### 4.4. Cell Cycle Analysis

Cells were seeded and subsequently treated with selected inhibitors over a period of 24 h. We then fixed the cells using cold 70% ethanol and treated them with ribonuclease. Cell cycle-dependent uptake of PI was observed using flow cytometry and cell cycle phases quantified using ModFit LT software version 3.2 (Verity Software House, Topsham, Maine, USA).

### 4.5. Oxygen Consumption

Cells were seeded in 24-well plates with integrated oxygen sensors and were exposed to the indicated inhibitors diluted in serum-enriched medium for 4 h. After renewal of the medium, the wells were covered with sterile paraffin oil to ensure airtight overlay. Incubated at 37.0 °C, fluorescence-based measurement of oxygen consumption was performed using the Oxo Dish OD24 (PreSens, Regensburg, Germany).

### 4.6. Glucose Consumption

Following pretreatment with inhibitors in serum-free medium for 4 h, cells were exposed to inhibitors diluted in serum-free medium with 2 mM glucose. The remaining glucose in the supernatant was analyzed after 8 h of treatment using the biochemistry analyzer Hitachi 917.

### 4.7. Reactive Oxygen Species Analysis

We performed reactive oxygen species (ROS) analysis as described previously [47]. Briefly, cells were treated as indicated in serum-free medium over 12 h. They were then washed with PBS and incubated for 30 min with 10 µM H_2_DCFDA (Invitrogen). Washed again with PBS, the cells were incubated with trypsine for 5 min and resuspended with PBS. Flow cytometry of DCF signal was performed with BD Canto II.

### 4.8. PTEN Gene Sequencing

DNA was purified using the PureLink Genomic DNA Kit (Thermo Fisher Scientific, Waltham, Massachusetts, USA) and sequenced by next generation sequencing by use of the GeneRad DNAseq Custom panel V3 (Qiagen, Venlo, Netherlands). The data were analyzed with QIAGEN Clinical Insight Analyze (QCIA) and QIAGEN Clinical Insight (QCI) Interpret. After filtering for artefacts and genomic variants, all somatic variants with allele frequencies of ≥5% were reported. For classification and interpretation of mutations, the following databases were used: dbSNP, COSMIC, gnomAD, ClinVar, OncoKB, CIVI. Regions investigated in the PTEN gene were exons 1, 2, 3, 4, 5, 6, 7, 8.

### 4.9. Statistical Analysis

All statistical analyses were performed with Grad Pad Prism 5.0 (GraphPad Software, Inc., San Diego, CA, USA). Quantitative data are expressed as indicated and include standard deviations (S.D.). The two-tailed Student’s *t*-test was used to determine *p*-values. A significance level of α = 0.05 was chosen for all tests.

## Figures and Tables

**Table 1:** Characteristic mutations of LN-308, LNT-229, G55 and T98G glioma cell lines. IDH1 = isocitrate dehydrogenase 1; MGMT = *O*-6-methylguanine-DNA methyltransferase gene promoter; PTEN = phosphatase and tensin homolog; WT = wildtype.

Cell Line	IDH1	MGMT	PTEN	P53
LN-308	WT	Methylated	Mutant	Deleted
LNT-229	WT	Methylated	WT	WT
G55	WT	Unmethylated	WT	Mutant
T98G	WT	Unmethylated	Mutant	Mutant

## Supplementary Materials

Supplementary materials can be found at https://www.mdpi.com/1422-0067/20/18/4474/s1.

## Abbreviations

CV	Crystal violet
FCS	Fetal calf serum
GB	Glioblastoma
PI	Propidium iodide
ROS	Reactive oxygen species
