# Supplementary material for: Second Generation mTOR Inhibitors as a Double-Edged Sword in Malignant Glioma Treatment
Source: Int J Mol Sci. 2019 Sep 10;20(18):4474. doi: 10.3390/ijms20184474 (PMC6770420; doi:10.3390/ijms20184474)
Supplement: Supplementary file 1 [file ijms-20-04474-s001.pdf]

International Journal of
Molecular Sciences

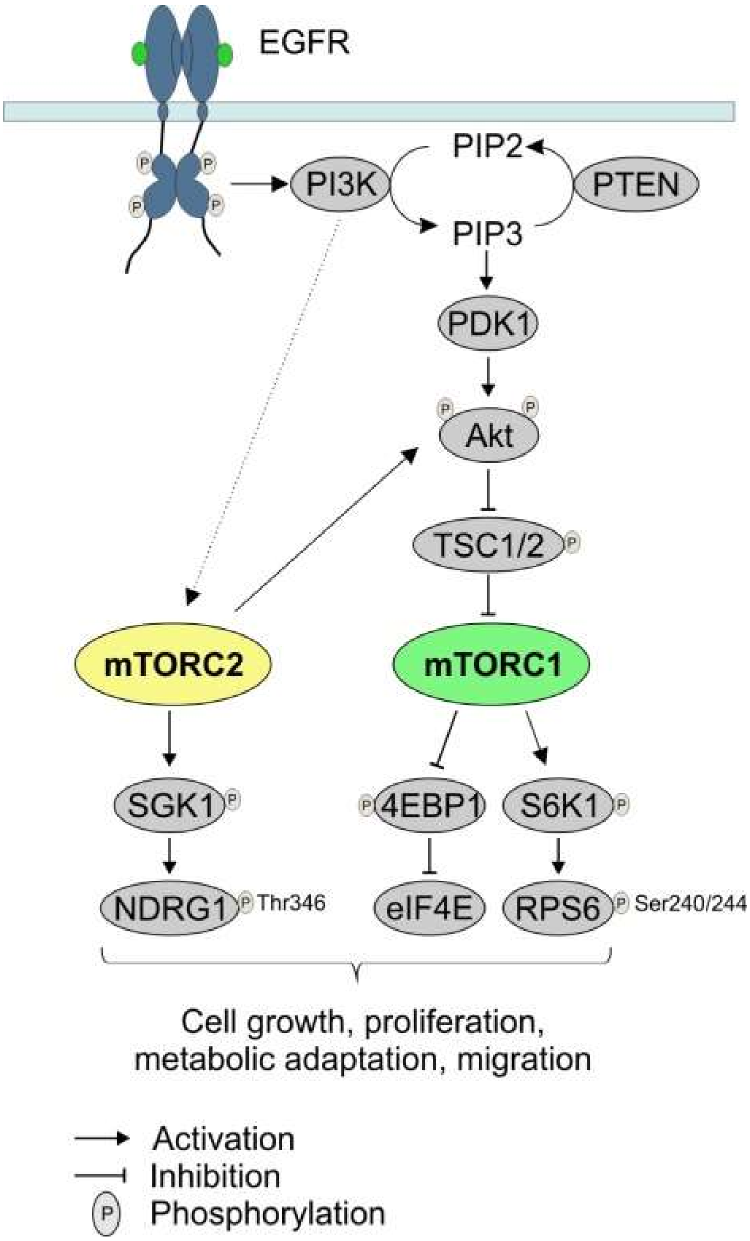

Figure S1. Schematic illustration of the epidermal growth factor receptor signaling cascade. Upon ligand binding or through genetic mutation, epidermal growth factor receptor (EGFR) signaling activates phopshoinositole-3-kinase (PI3K), which produces phosphatidylinositol-3,4,5-triphosphate (PIP3) by phosphorylating phosphatidylinositol-4,5-bisphosphate (PIP2). This conversion is reversed by the phosphatase and tensin homolog (PTEN). PIP3 binds to the 3-phosphoinositide-dependent protein kinase 1 (PDK1) which in turn activates Akt. Following phosphorylation of TSC1/2, inhibition of mTORC1 is relieved and then phosphorylates its two key downstream targets 4EBP1 and S6RP. While 4EBP1 inhibits the eukaryotic translation initiation factor 4E (eIF4E), S6RP signals via the ribosomal protein S6 (RPS6). PI3K also induces mTORC2 and leads to phosphorylation of the n-myc downstream regulated gene 1 (NDRG1) by the serine/threonine-protein kinase 1 (SGK1). Additionally, mTORC2 also phosphorylates Akt. Notably, mTORC2-mediated activation of Akt is not required for the phosphorylation of TSC1/2 [43].

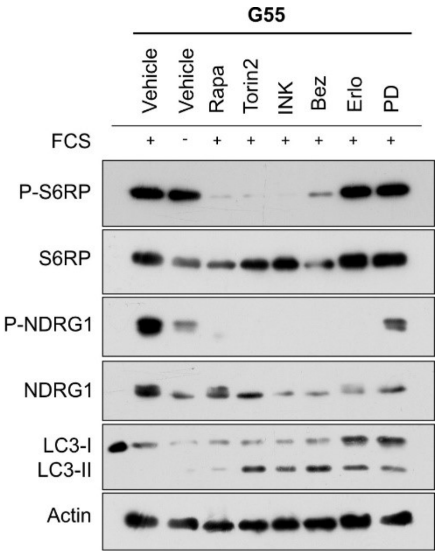

Figure S2. ATP-competitive mTOR inhibitors effectively inhibit mTORC1 and mTORC2 signal transduction in G55 glioma cells. G55 cells were treated for 2 h with rapamycin (100 nM), torin2 (100 nM), INK-128 (100 nM), NVP-Bez235 (10 nM), erlotinib (10 μM) or PD-153035 (10 μM). The levels of phosphorylated (P) and total S6RP and NDRG1, as well as LC3-B and actin, were assessed in cellular lysates by immunoblot.

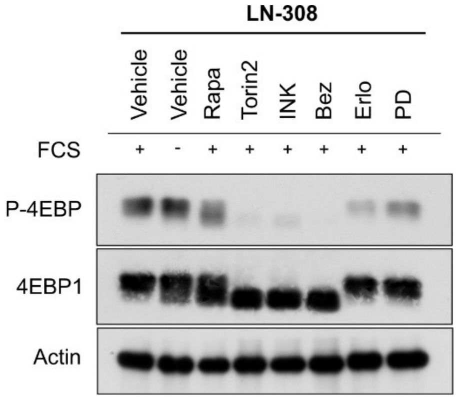

Figure S3. Dual mTORC1/2 inhibitors efficiently block phosphorylation of 4EBP1. LN-308 cells were treated for 2 h with rapamycin (100 nM), torin2 (100 nM), INK-128 (100 nM), NVP-Bez235 (10 nM), erlotinib (10 μM) or PD153035 (10 μM). Cellular lysates were analyzed by immunoblot with antibodies for phosphorylated (P) and total 4EBP1 or actin.

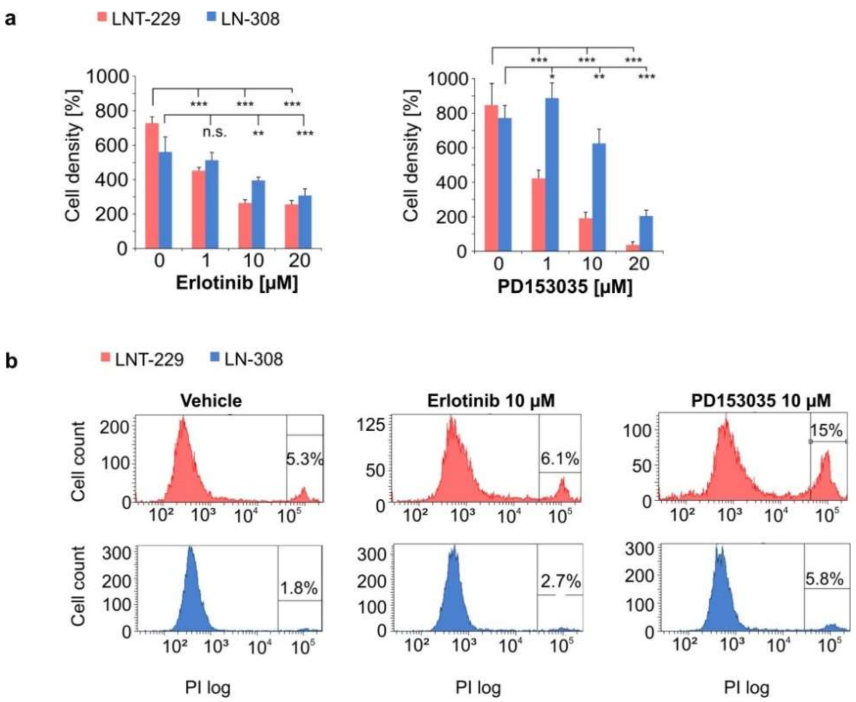

Figure S4. EGFR inhibitors reduce glioma cell growth and display cytotoxicity. (**a**) LNT-229 and LN-308 cells were treated with EGFR inhibitors erlotinib or PD153035 as indicated for 72 h. Cell density was analyzed by crystal violet staining. (n = 3, mean ± S.D.; *$p < 0.05$, **$p < 0.01$, ***$p < 0.001$, n.s. = not significant). (**b**) Cytotoxicity of erlotinib and PD153035 was assessed by propidiumiodide (PI) staining and consecutive FACS analysis after incubation for 72 h as described in (**a**) (one representative analysis of n = 3).

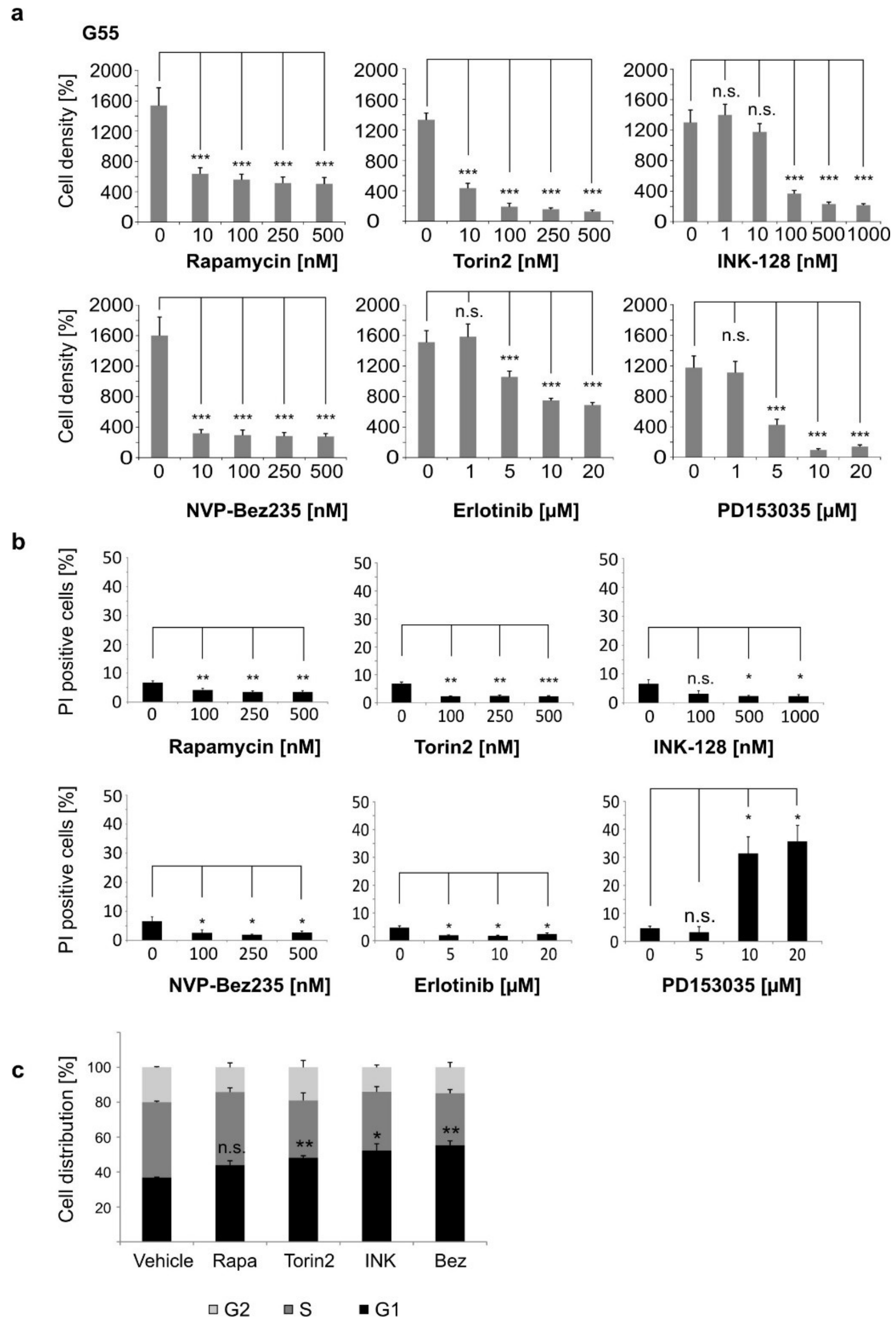

Figure S5. Inhibition of mTORC1 and 2 inhibits glioma cell growth of G55 cells by inducing cell cycle arrest. (**a**) G55 cells were incubated in medium containing 10% FCS and treated with rapamycin, torin2, INK-128, NVP-Bez235, erlotinib and PD153035 at the indicated concentrations for 72 h. Cell density was analyzed by crystal violet staining. Depicted values are normalized to cell density at the beginning of treatment ($n = 3$, mean ± S.D.; ***$p < 0.001$, n.s. = not significant). (**b**) Cytotoxicity was assessed by propidium iodide (PI) staining and consecutive FACS analyses ($n = 3$, mean ± S.D., *$p < 0.05$, **$p < 0.01$, ***$p < 0.001$, n.s. = not significant). (**c**) G55 cells were treated with the indicated EGFR or mTOR inhibitors for 24 h (100 nM rapamycin, 100 nM torin2, 100 nM INK-128, 10 nM NVP-Bez235, 10 µM erlotinib or 10 µM PD153035). DNA content as a marker of cell cycle phase was measured by PI staining after permeabilization using flow cytometry; results are shown as quantification of relative cell cycle phase distribution ($n = 3$, mean ± S.D., *$p < 0.05$, **$p < 0.01$, n.s. = not significant).

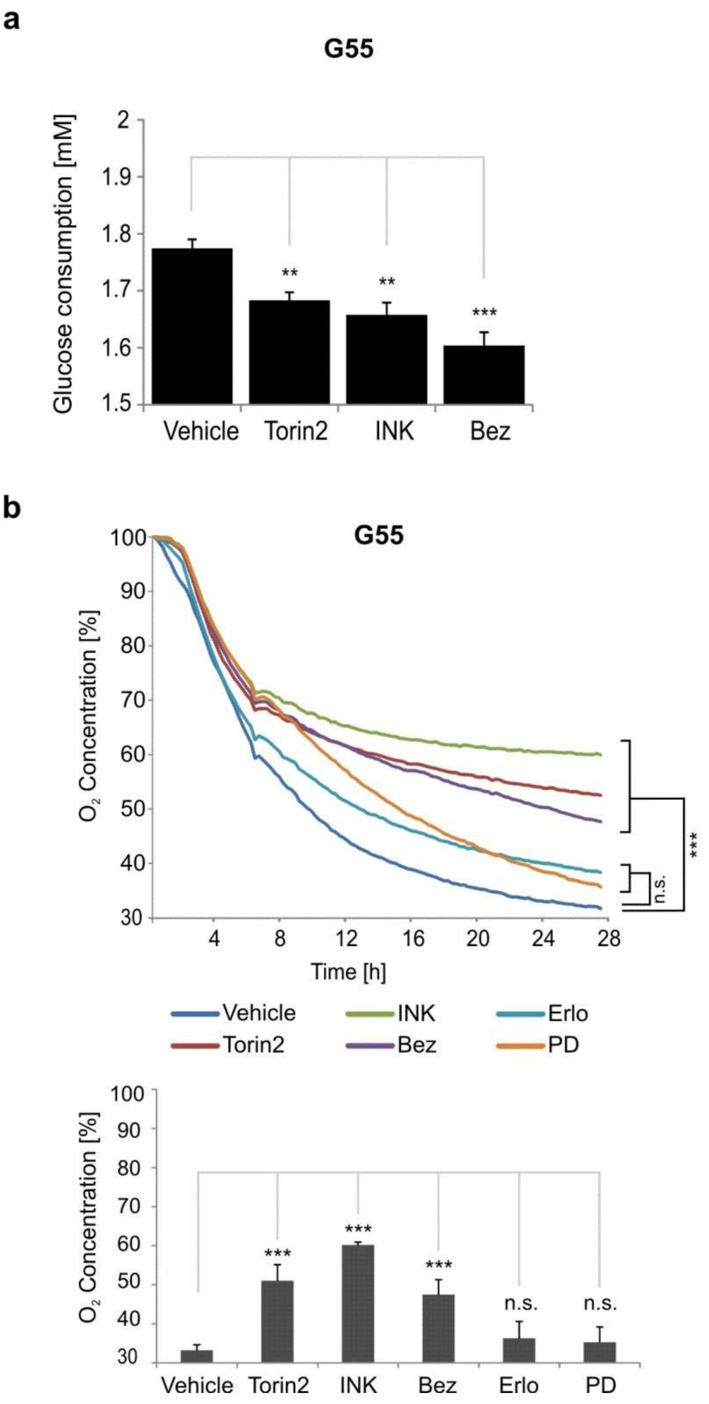

Figure S6. Inhibition of mTORC1 and 2 alters metabolism of G55 by reducing glucose and oxygen consumption. (**a**) G55 cells were cultured in medium containing 2 mM glucose in normoxia for 8 h. Glucose consumption was measured via quantification of remaining glucose in the supernant ($n = 3$, mean ± S.D.; $^{**}p < 0.01$ $^{***}p < 0.001$). (**b**) G55 cells were exposed to serum-free medium with 25 mM glucose and treated with rapamycin (100 nM), torin2 (100 nM), INK-128 (100 nM), NVP-Bez235 (10 nM), erlotinib (10 µM) and PD153035 (10 µM). Oxygen consumption was measured with a fluorescence-based assay ($n = 3$, mean; $^{***}p < 0.001$, n.s. = not significant). The end point analysis is depicted in the column charts ($n = 3$, mean ± S.D., $^{*}p < 0.05$, $^{**}p < 0.01$, $^{***}p < 0.001$).

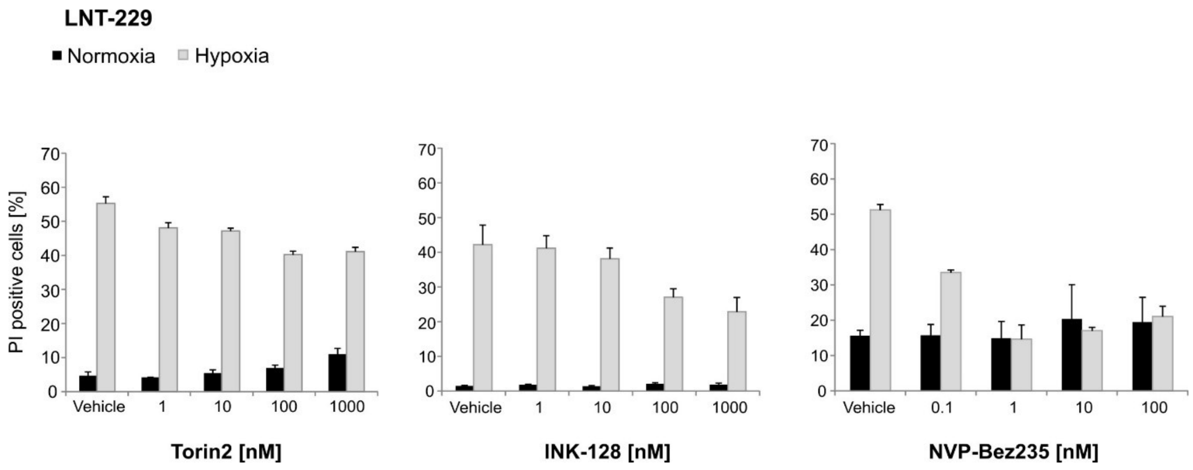

Figure S7. Dose-response curves of torin2, INK-128 and NVP-Bez235. LNT-229 cells were exposed to serum-free medium with 2 mM glucose and hypoxia (0.1% oxygen) and treated with torin2, INK-128 or NVP-Bez235 at indicated concentrations over 24–26 h. Cell death was then quantified by PI staining ($n = 3$, mean ± S.D.).

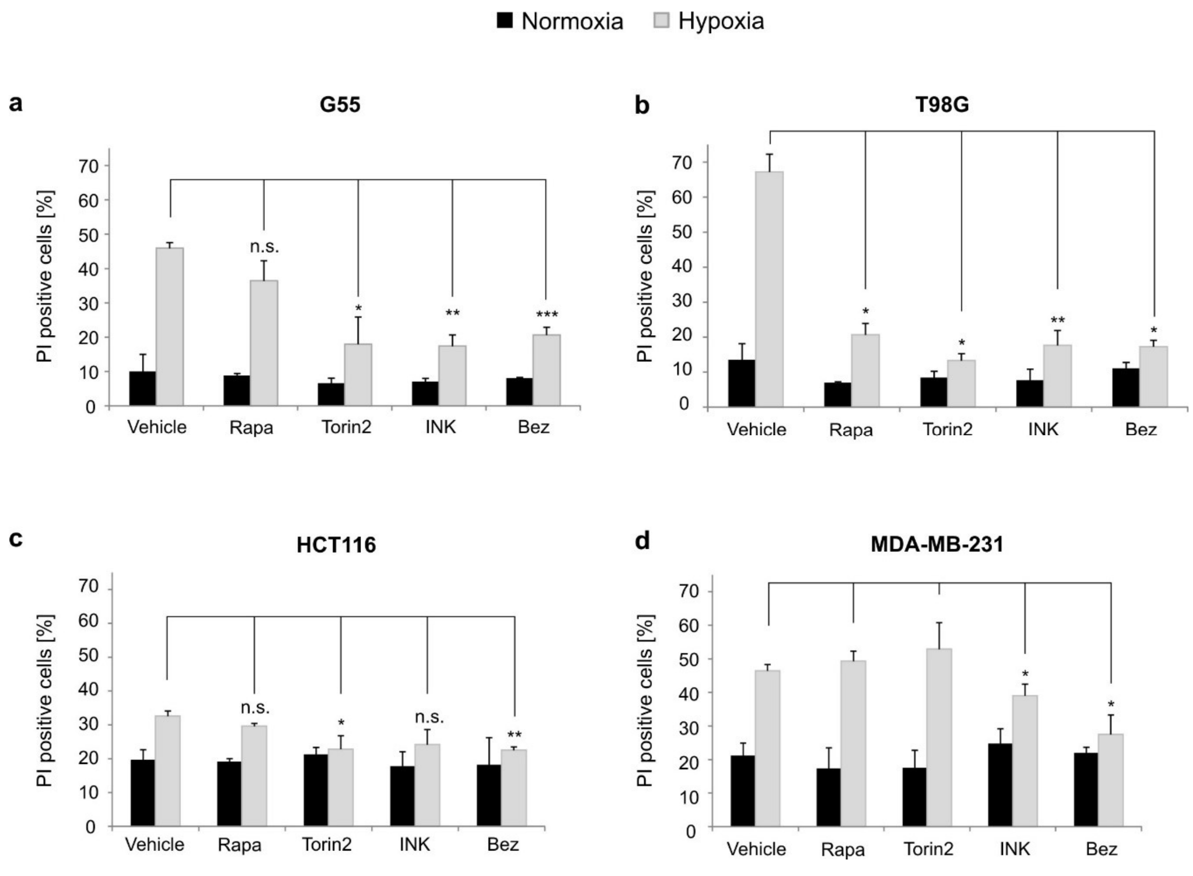

Figure S8. Inhibition of the mTOR pathway protects cell lines from different tumor entities from hypoxia-induced cell death. Glioma cell lines G55 (**a**) and T98G (**b**), as well as colon carcinoma cell line HCT116 (**c**) and breast carcinoma cell line MDA-MB-231 (**d**) were exposed to serum-free medium with 2 mM glucose and hypoxia (0.1% oxygen) and treated with rapamycin (100 nM), torin2 (100 nM), INK-128 (100 nM), NVP-Bez235 (10 nM), erlotinib (10 μM) or PD153035 (10 μM). Treatment duration was 26 h for G55 and T98G cells, 30 h for HCT116 and 40 h for MDA-MB-231 cells. Cell death was then quantified by PI staining ($n = 3$, mean ± S.D., *$p < 0.05$, **$p < 0.01$, ***$p < 0.001$).

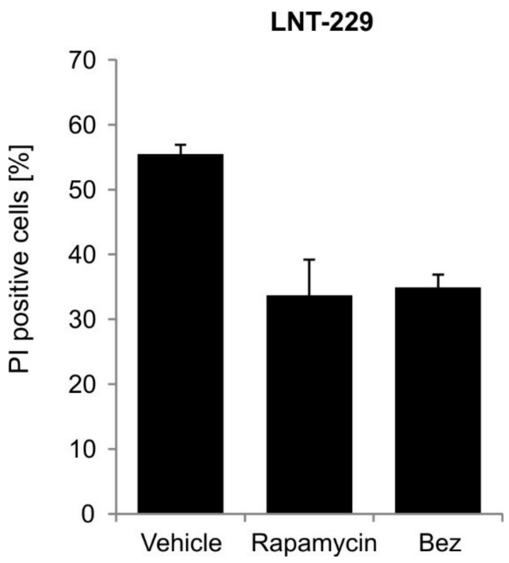

Figure S9. mTOR pathway inhibition protects LNT-229 cells from cell death associated with glucose restriction. LNT-229 cells were incubated in 21.0% oxygen and medium containing 2 mM glucose with vehicle, rapamycin (100 nM) or NVP-Bez235 (10 nM) for 96 h. Cell death was quantified by PI staining (n = 2, mean ± S.D.).

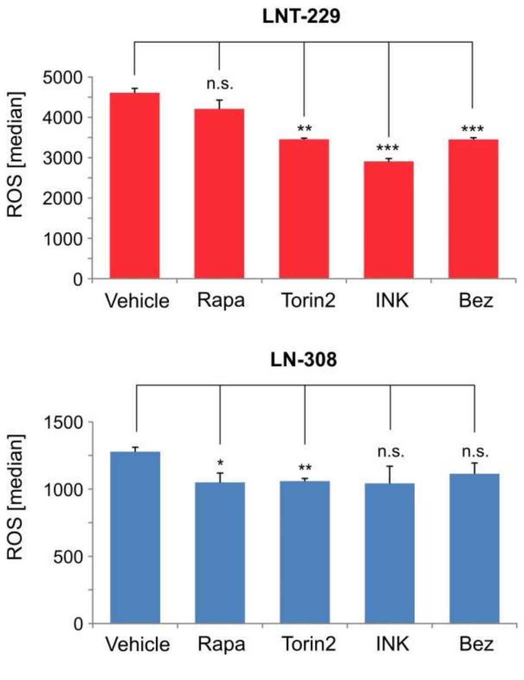

Figure S10. Inhibition of mTORC1 and 2 reduces levels of reactive oxygen species (ROS). Following treatment of LN-308 and LNT-229 cells with rapamycin (100 nM), torin2 (100 nM), INK-128 (100 nM) or NVP-Bez235 (10 nM) for 12 h, ROS levels were measured by H_2DCFDA-FACS (n = 3, mean ± S.D., *p < 0.05, **p < 0.01, ***p < 0.001, n.s. = not significant).

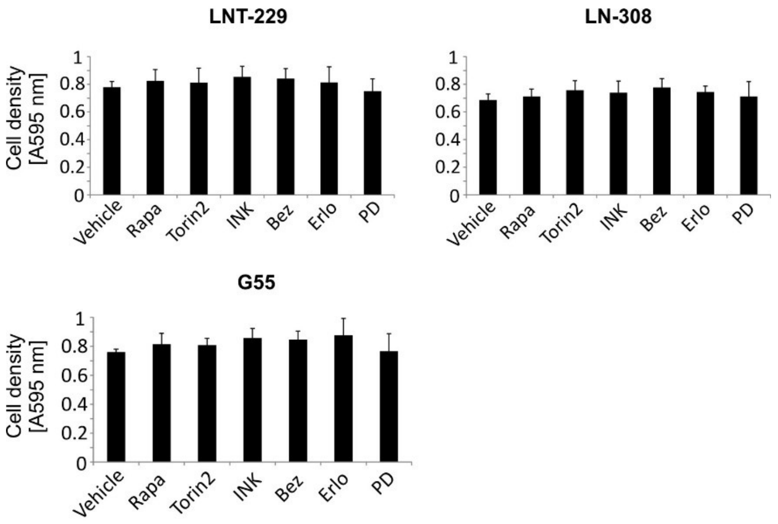

Figure S11. Brief preincubation with mTORC1/2 or EGFR inhibitors does not affect cell density. For measurement of glucose, oxygen consumption and hypoxia-induced cell death, LNT-229, LN-308 or G55 cells were preincubated with rapamycin (100 nM), torin2 (100 nM), INK-128 (100 nM), NVP-Bez235 (10 nM), erlotinib (10 μM) or PD153035 (10 μM) in serum-free medium for 4 h. Following preincubation, cell density was evaluated by CV staining (n = 3, mean ± S.D.).
